# Global real‐world data on hemodiafiltration: An opportunity to complement clinical trial evidence

**DOI:** 10.1111/sdi.13085

**Published:** 2022-04-19

**Authors:** Linda H. Ficociello, Ellen Busink, Dixie‐Ann Sawin, Anke Winter

**Affiliations:** ^1^ Global Medical Office Fresenius Medical Care Waltham MA USA; ^2^ Health Economics, Market Access and Political Affairs EMEA Fresenius Medical Care Deutschland GmbH Bad Homburg Germany; ^3^ Global Medical Office Fresenius Medical Care Bad Homburg Germany

## Abstract

Hemodiafiltration (HDF) is a renal replacement therapy that utilizes both diffusive clearance and convective transport to achieve greater clearance of middle‐molecular‐weight solutes. Among other factors, important prerequisites for the implementation of HDF include access to high‐flux dialyzers, achievement of high blood flow rates, and availability of high volumes of sterile substitution/replacement fluids. Online hemodiafiltration (OL‐HDF) is an established kidney replacement therapy, frequently used in many countries. Although in the United States, some prerequisites (e.g., access to high‐flux dialyzers and achievement of high blood flow rates) for OL‐HDF treatment are readily available; however, a machine capable of generating the online solution for OL‐HDF is currently not available. As the clinical experience with HDF accumulates globally, it is worth examining the evidence for this kidney replacement therapy as used in routine clinical care. Such real‐world evidence is increasingly recognized as valuable by clinicians and may inform regulatory decisions. In this review, we will focus on emerging global real‐world data derived from routine clinical practices and examine how these data may complement those derived from clinical trials.

## 1 INTRODUCTION

A number of kidney replacement therapies (KRT) have been developed for the long‐term management of patients with end‐stage kidney disease (ESKD). Broadly, hemodialysis can be classified as low‐flux, high‐flux, or hemodiafiltration (HDF).^1^ Low‐flux hemodialysis relies on diffusion to remove smaller uremic toxins. By using dialyzers with greater permeability, high‐flux hemodialysis applies diffusion and some convection to increase the clearance of molecules between 500 Da and 15 kDa but not larger middle molecules. HDF combines diffusion together with convective transport (secondary to filtration) to achieve greater clearance of middle‐molecular‐weight solutes.^2^, ^3^ As HDF involves the removal of large fluid volumes by convection, it requires equal large volumes of sterile substitution solution be infused during treatment. Historically, this fluid was transported and supplied in bags.^4^ Modern‐day HDF involves the production of ultrapure, sterile infusion solution by the dialysis machine at the bed side (i.e., online [OL]‐HDF). The use of HDF with large substitution volumes (high‐volume HDF; HVHDF) is variably defined as an effective convection volume of ≥20% of blood volume processed^5^ or convection/substitution volumes of >20–25 L/session.^6^, ^7^, ^8^, ^9^, ^10^

Due to regulatory barriers and the absence of available dialysis machines capable of OL‐HDF, HDF is rarely used in the United States.^4^, ^9^, ^11^ Nonetheless, OL‐HDF accounts for approximately 10% of global dialysis sessions.^12^, ^13^ As clinical experience with OL‐HDF accumulates, it is worth reexamining the clinical data for this modality of KRT. In this review, we focus on emerging data derived from routine clinical practice and examine how these data may complement those derived from clinical trials and aid clinical decision making.

## 2 THE ROLE OF REAL‐WORLD EVIDENCE IN ASSESSING THE CLINICAL PROFILE OF THERAPY

The ability to incorporate novel therapeutic approaches into routine clinical practice is generally dictated by well‐defined and mostly country‐specific regulatory pathways. Traditionally, the clinical profile (i.e., safety and efficacy) of therapeutic approaches is generated by well‐controlled randomized clinical trials. The data collected from such trials are frequently used to determine initial market authorization. However, these randomized clinical efficacy trials often provide little insight into the effectiveness and value of therapeutic approaches when used among broad, unselected patient populations in real‐world healthcare settings.^14^, ^15^ Thus, the need for real‐world evidence (RWE) to complement the results of traditional clinical trials is recognized by many healthcare stakeholders, as it can expand our knowledge and understanding of the risks, benefits, and value of therapeutic approaches when used in routine practice.^16^, ^17^ As will be reviewed, RWE can be generated by a variety of study designs and frameworks using diverse data sources.^18^

Interventional effectiveness trials randomly assign patients to one of several therapeutic approaches. The random treatment assignment enhances the internal validity of the study results, and additional design choices (e.g., selection of patient population, comparator, outcomes/study endpoints, duration of follow‐up, and overall setting) allow tailoring of the trial toward its overall objective of demonstrating effectiveness of a therapeutic intervention, within the constraints of conducting a trial. An alternative research framework to generate RWE uses observational studies of real‐world data (RWD). Observational studies can be designed such that the data collection is directed toward a specific research purpose (e.g., a prospective registry study). Alternatively, retrospective analyses of existing RWD sources (e.g., claims data or data stemming from electronic health records) can be analyzed. In such studies, treatment decisions are made by healthcare professionals and patients (i.e., not dictated by a study protocol), allowing a better understanding of current practice patterns. The inclusion of an unselected patient population such as national registries improves the generalizability of the results. Analysis of large, unrestricted patient populations also allows for investigation of rare outcome events, exploration of patient subgroups and accounts for various regional or local practices. Evidence from RWD sources can be generated in a timely and resource‐considerate fashion.

Interpretation of observational study results must be put into appropriate context. Specifically, the lack of random treatment assignment may introduce biases, particularly when attempting to compare different therapeutic approaches. Design and analytical strategies (e.g., restriction of patients, proper selection of unexposed patients through direct matching or propensity‐score matching (PSM), inverse probability of censoring weighting, using instrumental variables, or utilizing proxy measures for variables of interest) can reduce, but not eliminate, the possibility of confounding and bias.^19^ When analyzing data not collected for research purposes, data quality and completeness can vary greatly, a factor that should be considered when interpreting analysis results.

## 3 EVIDENCE BASE FOR HDF: RANDOMIZED CONTROLLED TRIALS (RCTS)

Data examining OL‐HDF have come from both RCTs and observational RWD generation. To date, four RCTs have investigated the effects of OL‐HDF on mortality risks or rates in different countries (the Dutch Convective Transport Study [CONTRAST],^12^ the Estudio de Supervivencia de Hemodiafiltración Online [ESHOL] study,^10^ the Comparison of Post‐dilution Online Haemodiafiltration and Haemodialysis [Turkish OL‐HDF] Study,^20^ and the French Convective versus Hemodialysis in Elderly [FRENCHIE] study^21^). However, data from the individual studies are conflicting and inconclusive, with only one of the four showing a beneficial effect on mortality. The ESHOL study compared the effects of HV OL‐HDF and high‐flux hemodialysis on all‐cause and cardiovascular mortality in approximately 600 patients over 24 months. The target convective volume (CV) was ≥18 L/session plus the net UF volume needed to achieve the patient's target or dry weight, and the delivered CV was 22.9–23.9 L/session. Mean blood flow rates (Qb) were >387 mL/min, and mean treatment duration was 236 min. OL‐HDF was associated with reduced risk of all‐cause mortality (30% relative risk reduction [RRR]), cardiovascular mortality (33% RRR), and hospitalization (22% RRR).^10^, ^22^ In those who received CVs > 25 L/session, all‐cause mortality was reduced by 45%.^10^, ^22^ Medication usage did not differ between groups. Notably, 6% of patients used low‐flux dialyzers, and the HDF group was younger, had a lower prevalence of diabetes, and had less severe comorbid disease (as assessed by lower Charlson Comorbidity Index scores), and fewer patients dialyzed with venous catheters.

In the Dutch CONTRAST, OL‐HDF with high‐flux dialyzers was compared with low‐flux hemodialysis among approximately 700 patients.^12^ No mortality benefit was observed with OL‐HDF over the 36‐month study period.^12^ Among patients in the OL‐HDF cohort, the mean (SD) filtration fraction was 25.9 (3.9) L (not exceeding 33%); Qb, 333 (44) mL/min; treatment duration, 225 (24) min; and target CV, 24 L/session (6 L/h).^23^ Although the target CV was 24 L/session (6 L/h), 50–66% of patients did not achieve this target, and the mean delivered CV was below the target at 20.7 L/session. Notably, patients who received >21.95 L of CV (assuming 2 L of UF needed to achieve the patient's target weight) with OL‐HDF showed a significantly lower mortality compared to those randomized to low‐flux HD. The target CV was reached in only 18% of these patients, who incidentally had higher Qb (384 ± 5 mL/min).^23^

The Turkish OL‐HDF Study aimed to compare the effects of OL‐HDF on all‐cause mortality and first nonfatal cardiovascular event requiring hospitalization with those of high‐flux hemodialysis.^20^ During OL‐HDF sessions, the filtration fraction was kept between 25% and 30%, and the substitution volume was targeted to be >15 L and Qb of 250–400 mL/min. In the OL‐HDF group, the achieved CV was 17.2 L/session and mean treatment duration 236 min. Cumulative event‐free survival, cardiovascular and overall survival, hospitalization rates, number of hypertensive episodes, and medication use did not differ between treatment groups. In post hoc analyses, substitution volumes >17.4 L/session were associated with a 30% reduced risk of the primary outcome and a 46% reduced risk of all‐cause mortality. Notably, 10% (*n* = 40) of the patients in the OL‐HDF group terminated the study early due to vascular access problems, predominantly secondary to insufficient Qd.

Most recently, the FRENCHIE study assessed high‐flux conventional hemodialysis versus OL‐HDF among 381 patients over 65 years of age.^21^ Treatments were conducted thrice‐weekly for approximately 237 min/session. Target rates were 350–400 mL/min (Qb) and 500–600 mL/min (Qd). Relative to high‐flux hemodialysis, OL‐HDF was not associated with any significant effect on all‐cause or cardiovascular mortality. However, OL‐HDF was associated with fewer episodes of intradialytic hypotension, a higher rate of arrhythmias, and a 47% reduced risk of vascular access hospitalizations in this elderly group. Apart from the mental composite score of the Kidney Disease Quality of Life Short Form (KDQOL‐SF) questionnaire, which was higher in the conventional HD group, patient‐reported quality of life did not differ between groups. Since the primary endpoint of the study was intradialytic tolerance and not mortality, the study was underpowered to assess mortality differences between the two dialysis modalities, and mortality in the ESKD study population was low.

Meta‐analyses of available data, by both Mostovaya et al. and Nistor et al., reported decreased risk of cardiovascular mortality with HDF relative to hemodialysis (relative risk [RR]: 0.73 and 0.75, respectively).^24^, ^25^ In an assessment of mortality among patients from all four RCTs (*N* = 2793), Nubé et al. reported that the beneficial effect of OL‐HDF on survival seen in the overall analysis by Peters et al. was due to the effect of lowering the risk of cardiac death (hazard ratio [HR] [95% CI]: 0.64 [0.61; 0.90], p = 0.01).^26^, ^27^

A fifth RCT, the Impact of Hemodiafiltration on Physical Activity and Self‐Reported Outcomes (HDFIT) trial, compared high‐flux hemodialysis versus HVHDF but did not examine so‐called “hard” clinical outcomes such as mortality and hospitalization.^28^ Instead, the trial evaluated the impact of HDF on overall patient‐reported physical activity and sleep duration in 13 Brazilian dialysis clinics. Investigators found no effect of dialysis modality on sleep duration. Independent of modality, patients receiving dialysis between 6 am and 10 am (i.e., the first shift) experienced significant reductions in sleep duration.^28^ In a meta‐analysis of five RCTs (*N* = 1259), Wang and colleagues (*N* = 1259) found that HDF reduced symptomatic hypotension (RR [95% CI]: 0.49 [0.30; 0.81]; p = 0.002).^29^ Discordance among the above RCTs may stem from multiple factors, including study designs, choice of comparators or control groups, selection bias, and the presence of confounders such as use of low‐flux HD membranes, differences in substitution volume targets, failure of patients to reach target volumes/rates, and/or inconsistency in CVs delivered.

The ongoing CONVINCE study is an RCT that aims to address some of the shortcomings of prior trials.^30^ By aiming to enroll 1800 patients, the hypothesis that treatment with OL‐HDF, when consistently delivered in high doses of >23 L of substitution volume, results in an improvement in clinical outcomes will be tested over a 3‐year follow‐up period. Study endpoints include all‐cause and cause‐specific mortality, cardiovascular morbidity, and various patient‐reported outcomes.^31^

## 4 EVIDENCE BASE FOR HDF: OBSERVATIONAL STUDIES

Although a beneficial effect of OL‐HDF on mortality has not been conclusively demonstrated in RCTs, several observational studies strongly suggest a survival benefit afforded by HD OL‐HDF. Data from the Dialysis Outcomes and Practice Patterns Study (DOPPS) that followed more than 2100 patients revealed a 35% reduced risk of mortality (RR: 0.65) compared with high‐ (RR: 1.03) or low‐flux HD (reference) among patients receiving OL‐HDF with CVs of 15–24.9 L/session.^32^ However, a second DOPPS RWD analysis did not find any survival benefit with OL‐HDF with substitution volumes >20 L.^33^ Interpretation of the findings is tempered by the fact that only 6% of participating clinics prescribed OL‐HDF to all patients; when the analysis was adjusted for study era and country, a CV > 20 L was associated with a 10% reduction in all‐cause mortality. When results were further adjusted for vascular access, Qb, body mass index, hemoglobin, and serum albumin, conventional HD appeared to confer a survival benefit (relative to facilities with higher rates of OL‐HDF). Discrepancies between the two DOPPS data analyses may be attributed to achieved CVs, study design confounders, center effects, actual numbers of patients prescribed OL‐HDF, and sampled populations.

Data from the European Clinical Database (EuCliD) on 394 patients receiving OL‐HDF and 2170 receiving hemodialysis were examined.^34^ Investigators demonstrated a 35.3% reduced risk of mortality associated with HD OL‐HDF (15–25 L/session) relative to hemodialysis.^35^ The RISCAVID (RISchio CArdiovascolare nei pazienti afferenti all' Area Vasta In Dialisi) study examined data from 757 patients with ESKD and found that OL‐HDF (22–25 L/session of CV) resulted in higher cumulative survival than standard HD, even after model adjustments (RR for mortality: 0.78; p = 0.01).^36^ In 2018, See and colleagues published data from the Australia and New Zealand Dialysis and Transplant (ANZDATA) Registry.^37^ In a cohort of nearly 23,000 patients receiving hemodialysis and more than 4000 patients receiving OL‐HDF, investigators demonstrated a mortality benefit with OL‐HDF. Multivariate analyses demonstrated 21% and 12% RRRs in all‐cause mortality in Australia (*N* = 3302; p < 0.001) and New Zealand (*N* = 808; p = 0.05), respectively. A significant treatment effect on cardiovascular mortality was observed in the Australian cohort (HR: 0.79; p = 0.01) but not the New Zealand cohort (HR: 1.09; p = 0.48). Qb ranged from <250 to >350 mL/min, and CVs were <17 to >22 L/session. A majority (60%) of patients permanently remained on OL‐HDF, and of the 40% who discontinued OL‐HDF, more than a quarter (28%) reinitiated OL‐HDF at a later time. A 2019 study from the Japanese Society for Dialysis Therapy Renal Data Registry examined 1‐year survival outcomes in a propensity‐matched cohort of 5000 pairs of patients treated with high‐flux conventional hemodialysis or predilution OL‐HDF.^38^ OL‐HDF was associated with improved overall survival (HR [95% CI] for all cause‐mortality: 0.83 [0.705; 0.986]), with a trend toward improved cardiovascular survival.

The potential benefits of OL‐HDF appear to extend to patients first starting dialysis (i.e., incident patients). For instance, a significant reduction (71%) in mortality was also associated with HD (≥20.4 L) OL‐HDF (vs. high‐flux conventional hemodialysis) in an observational trial among 442 incident ESKD patients (HR: 0.29).^39^ In a separate study of nearly 1600 incident patients, HVHDF was associated with a 50% reduction in mortality relative to hemodialysis.^40^ Subgroup analyses further supported a potential survival benefit with higher CVs in patients aged 65–74 years, females, obese patients, non‐diabetic patients, and patients with high blood pressure. In a PSM cohort study by Maduell et al. among approximately 1000 incident patients across 64 Spanish dialysis clinics, OL‐HDF (median SV: 23.45 L) was associated with a 24% RRR in all‐cause mortality.^41^ A significant reduction in the risk of cardiovascular mortality was also observed with OL‐HDF (HR: 0.67; p = 0.008).

## 5 PERSPECTIVES ON RWE

The last 20 years have seen an exponential growth in the number of articles on RWE. The onset of the coronavirus disease 2019 (COVID‐19) pandemic has put an even greater focus on RWE as the collection of clinical trial data is now even more challenging.^42^ Despite such focus, the role of RWE in decision‐making processes for (a) regulators, (b) clinicians, and (c) payers and health technology assessment (HTA) agencies remain mixed.

The European Medicines Agency (EMA) introduced strategies incorporating RWE for early access to medicines addressing high unmet medical needs. Those medicines with insufficient “traditional data” may be approved for use with the understanding that further RWE will be collected (e.g., through product registries, claims data, and electronic health records) once the therapy is in use.^43^ Presently, RWE has been given even more prominence in the approval process for medical devices since the introduction of the new Medical Device Regulation in the European Union.^44^

The role of RWE in the approval process of therapeutics appears to be increasing. In fact, in 2018 and 2019, 40% of initial marketing authorization applications included RWE.^45^ In the Pharmaceutical Strategy for Europe, adopted by the European Union in 2020, “big and real‐world data” will “support the development, authorization and use of medicines” by 2025^46^, ^47^; a key component aimed at advancing the use of RWE is the Data Analytics and Real‐World Interrogation Network (DARWIN EU), slated to launch in 2022.^48^

There are few data examining the extent to which clinicians use RWE to guide treatment decisions. In a survey of U.S. oncologists, more than three‐quarters of those surveyed indicated RWE is necessary to inform clinical decisions, but nearly 70% denied using RWE in such decision making.^49^ In another survey, 58% of U.S. cardiologists indicated that RWE can be used to “tailor health care decisions more closely to the characteristics of individual patients.”^50^ Clinicians see RWE as a useful complementary source to traditional forms of data collection, especially with regard to safety and effectiveness among heterogeneous populations not included in other trials and for outcome parameters not previously examined.^51^ It is reasonable to assume that clinician trust in, and reliance on, RWE will increase as the reliability of such data increases.

Payers have recognized the potential value of RWE, especially with regard to reimbursement for highly innovative technologies.^52^ However, the use of RWE for decision making remains limited for this stakeholder group. In a review of 27 pharmacy and therapeutic committee monographs for U.S. payers, RWE accounted for less than 5% of the data sources.^53^ The literature does point to a trend for this to change in the near future, as a result of revised guidance and steps to improve the quality of RWE.^54^, ^55^, ^56^, ^57^

## 6 RWE FOR DECISION MAKING RELATED TO OL‐HDF

A large body of RWE has been generated for OL‐HDF, and these data complement the body of evidence from RCTs. The available data suggest that the clinical benefits observed in some controlled trials are also demonstrated in a real‐world setting. To date, we are unaware of any RWE generated for OL‐HDF having been used for regulatory authorization or payor decisions. The body of published RWE, particularly from Europe, has increased over time, suggesting clinical experience and comfort levels with this modality of dialysis are rising.

Examples of currently ongoing effectiveness trials comparing HVHDF and high‐flux hemodialysis are the CONVINCE trial and the U.K. High‐volume Haemodiafiltration versus High‐flux Haemodialysis Registry Trial (H4RT).^30^, ^58^ Both trials have mortality as their primary outcome measures but have also included patient‐reported outcomes and economic evaluation in their trial designs. The evidence generated through these trials holds the promise of answering important questions regarding the benefits and risks of OL‐HDF when used in real‐world practice. Both trials will also allow us to examine health economic outcomes, enabling a better understanding of the value of OL‐HDF. Despite some of the methodological drawbacks, observational research studies based on RWD can provide valuable and important evidence on real‐world utilization and practice patterns among unselected patient populations and complement the results of clinical trials.

Whereas in‐center OL‐HDF is essentially nonexistent in the United States, approximately 23% of European dialysis patients in 2012 were treated with OL‐HDF, with a high variation between individual countries.^33^ DOPPS data from the Middle East suggest OL‐HDF are being used for approximately 20% of patients—with again considerable variation by country and gender (men: 23%; women: 16%).^59^ Dissemination of these experiences, along with published RCTs and RWD studies, may help inform U.S. nephrologists. Early adopters, including physicians with OL‐HDF experience who have practiced outside the United States, may help to lead the paradigm shift to treating patients with OL‐HDF, but education on the therapy and prescriptions will need to be conducted. There is some hesitancy over OL‐HDF among U.S. nephrologists; the most frequently cited concerns are complexity, expense, and seemingly inconsistent patient outcomes.^4^ The capability of current dialysis machines to generate substitution fluid OL, rather than rely on pre‐packaged bagged solutions, removes much of the complexity and additional cost.^60^ No additional staffing is needed to run OL‐HDF treatments, although staff training would be required, just as with any new machine. Clinical outcomes, as reviewed above, are more consistent when studies focus on HVHDF. Therefore, HVHDF is recommended over OL‐HDF with lower substitution volumes.^61^ With the potential savings from OL solution generation and improved patient outcomes, the argument can be made that OL‐HDF offers increased value for both patients and payors.

## References

[1] Locatelli F , Carfagna F , del Vecchio L , La Milia V . Haemodialysis or haemodiafiltration: that is the question. Nephrol Dial Transplant. 2018;33(11):1896‐1904. doi:10.1093/ndt/gfy035 29688552

[2] Ward RA , Schmidt B , Hullin J , Hillebrand GF , Samtleben W . A comparison of on‐line hemodiafiltration and high‐flux hemodialysis: a prospective clinical study. J Am Soc Nephrol. 2000;11(12):2344‐2350. doi:10.1681/ASN.V11122344 11095657

[3] Haroon S , Davenport A . Choosing a dialyzer: what clinicians need to know. Hemodial Int. 2018;22(S2):S65‐s74. doi:10.1111/hdi.12702 30296005

[4] Blankestijn PJ . Has the time now come to more widely accept hemodiafiltration in the United States? J Am Soc Nephrol. 2013;24(3):332‐334. 23393321 10.1681/ASN.2013010063

[5] Tattersall JE , Ward RA . Online haemodiafiltration: definition, dose quantification and safety revisited. Nephrol Dial Transplant. 2013;28(3):542‐550. doi:10.1093/ndt/gfs530 23345621

[6] Panichi V , Scatena A , Rosati A , et al. High‐volume online haemodiafiltration improves erythropoiesis‐stimulating agent (ESA) resistance in comparison with low‐flux bicarbonate dialysis: results of the REDERT study. Nephrol Dial Transplant. 2015;30(4):682‐689. doi:10.1093/ndt/gfu345 25385719

[7] Schiffl H . High‐volume online haemodiafiltration treatment and outcome of end‐stage renal disease patients: more than one mode. Int Urol Nephrol. 2020;52(8):1501‐1506. doi:10.1007/s11255-020-02489-9 32488753 PMC7378113

[8] Bowry SK , Canaud B . Achieving high convective volumes in on‐line hemodiafiltration. Blood Purif. 2013;35(Suppl 1):23‐28. doi:10.1159/000346379 23466374

[9] Schiffl H . Online hemodiafiltration and mortality risk in end‐stage renal disease patients: a critical appraisal of current evidence. Kidney Res Clin Pract. 2019;38(2):159‐168. doi:10.23876/j.krcp.18.0160 31137926 PMC6577208

[10] Maduell F , Moreso F , Pons M , et al. High‐efficiency postdilution online hemodiafiltration reduces all‐cause mortality in hemodialysis patients. J Am Soc Nephrol. 2013;24(3):487‐497. doi:10.1681/ASN.2012080875 23411788 PMC3582206

[11] Golper TA . Is hemodiafiltration ready for broader use? Kidney Int. 2015;88(5):940‐942. 26579679 10.1038/ki.2015.251

[12] Grooteman MP , van den Dorpel MA , Bots ML , et al. Effect of online hemodiafiltration on all‐cause mortality and cardiovascular outcomes. J Am Soc Nephrol. 2012;23(6):1087‐1096. doi:10.1681/ASN.2011121140 22539829 PMC3358764

[13] Ward RA , Vienken J , Silverstein DM , Ash S , Canaud B . Regulatory Considerations for Hemodiafiltration in the United States. Clin J Am Soc Nephrol. 2018;13(9):1444‐1449. doi:10.2215/CJN.12641117 29511058 PMC6140579

[14] Kitchlu A , Shapiro J , Amir E , et al. Representation of patients with chronic kidney disease in trials of cancer therapy. Jama. 2018;319(23):2437‐2439. doi:10.1001/jama.2018.7260 29922818 PMC6583039

[15] Konstantinidis I , Patel S , Camargo M , et al. Representation and reporting of kidney disease in cerebrovascular disease: a systematic review of randomized controlled trials. PLoS ONE. 2017;12(4):e0176145. doi:10.1371/journal.pone.0176145 28426831 PMC5398672

[16] O'Hare AM , Rodriguez RA , Bowling CB . Caring for patients with kidney disease: shifting the paradigm from evidence‐based medicine to patient‐centered care. Nephrol Dial Transplant. 2016;31(3):368‐375. doi:10.1093/ndt/gfv003 25637639 PMC4762396

[17] Sherman RE , Anderson SA , Dal Pan GJ , et al. Real‐World evidence—what is it and what can it tell us? N Engl J Med. 2016;375(23):2293‐2297. doi:10.1056/NEJMsb1609216 27959688

[18] Orsini LS , Berger M , Crown W , et al. Improving transparency to build trust in real‐world secondary data studies for hypothesis testing‐why, what, and how: recommendations and a road map from the real‐world evidence transparency initiative. Value Health. 2020;23(9):1128‐1136. doi:10.1016/j.jval.2020.04.002 32940229

[19] Brookhart MA , Stürmer T , Glynn RJ , Rassen J , Schneeweiss S . Confounding control in healthcare database research: challenges and potential approaches. Med Care. 2010;48(6 Suppl):S114‐S120. doi:10.1097/MLR.0b013e3181dbebe3 20473199 PMC4024462

[20] Ok E , Asci G , Toz H , et al. On behalf of the 'Turkish Online Haemodiafiltration Study'Mortality and cardiovascular events in online haemodiafiltration (OL‐HDF) compared with high‐flux dialysis: results from the Turkish OL‐HDF Study. Nephrol Dial Transplant. 2013;28(1):192‐202. doi:10.1093/ndt/gfs407 23229932

[21] Morena M , Jaussent A , Chalabi L , et al. Treatment tolerance and patient‐reported outcomes favor online hemodiafiltration compared to high‐flux hemodialysis in the elderly. Kidney Int. 2017;91(6):1495‐1509. doi:10.1016/j.kint.2017.01.013 28318624

[22] Maduell F , Moreso F , Mora‐Macià J , et al. ESHOL study reanalysis: all‐cause mortality considered by competing risks and time‐dependent covariates for renal transplantation. Nefrologia. 2016;36(2):156‐163. doi:10.1016/j.nefro.2015.10.007 26672890

[23] Penne EL , van der Weerd NC , Bots ML , et al. Patient‐ and treatment‐related determinants of convective volume in post‐dilution haemodiafiltration in clinical practice. Nephrol Dial Transplant. 2009;24(11):3493‐3499. 19515802 10.1093/ndt/gfp265

[24] Mostovaya IM , Blankestijn PJ , Bots ML , et al. Clinical evidence on hemodiafiltration: a systematic review and a meta‐analysis. Semin Dial. 2014;27(2):119‐127. doi:10.1111/sdi.12200 24738146

[25] Nistor I , Palmer SC , Craig JC , et al. Haemodiafiltration, haemofiltration and haemodialysis for end‐stage kidney disease. Cochrane Database Syst Rev. 2015;(5):Cd006258. doi:10.1002/14651858.CD006258.pub2 25993563 PMC10766139

[26] Peters SA , Bots ML , Canaud B , et al. Haemodiafiltration and mortality in end‐stage kidney disease patients: a pooled individual participant data analysis from four randomized controlled trials. Nephrol Dial Transplant. 2016;31(6):978‐984. 26492924 10.1093/ndt/gfv349

[27] Nubé MJ , Peters SAE , Blankestijn PJ , et al. Mortality reduction by post‐dilution online‐haemodiafiltration: a cause‐specific analysis. Nephrol Dial Transplant. 2017;32(3):548‐555. 28025382 10.1093/ndt/gfw381

[28] Han M , Guedes M , Larkin J , et al. Effect of hemodiafiltration on self‐reported sleep duration: results from a randomized controlled trial. Blood Purif. 2020;49(1–2):168‐177. doi:10.1159/000504242 31851982

[29] Wang AY , Ninomiya T , Al‐Kahwa A , et al. Effect of hemodiafiltration or hemofiltration compared with hemodialysis on mortality and cardiovascular disease in chronic kidney failure: a systematic review and meta‐analysis of randomized trials. Am J Kidney Dis. 2014;63(6):968‐978. doi:10.1053/j.ajkd.2014.01.435 24685515

[30] Blankestijn PJ , Fischer KI , Barth C , et al. Benefits and harms of high‐dose haemodiafiltration versus high‐flux haemodialysis: the comparison of high‐dose haemodiafiltration with high‐flux haemodialysis (CONVINCE) trial protocol. BMJ Open. 2020;10(2):e033228. 10.1136/bmjopen-2019-033228 PMC7044930 32029487

[31] Netherlands Trials Register: CONVINCE. Trial NL6942 (NTR7138). December 21, 2021. https://www.trialregister.nl/trial/6942

[32] Canaud B , Bragg‐Gresham JL , Marshall MR , et al. Mortality risk for patients receiving hemodiafiltration versus hemodialysis: European results from the DOPPS. Kidney Int. 2006;69(11):2087‐2093. doi:10.1038/sj.ki.5000447 16641921

[33] Locatelli F , Karaboyas A , Pisoni RL , et al. Mortality risk in patients on hemodiafiltration versus hemodialysis: a ‘real‐world’ comparison from the DOPPS. Nephrol Dial Transplant. 2018;33(4):683‐689. doi:10.1093/ndt/gfx277 29040687 PMC5888924

[34] Jirka T , Cesare S , di Benedetto A , et al. Mortality risk for patients receiving hemodiafiltration versus hemodialysis. Kidney Int. 2006;70(8):1524‐1525. doi:10.1038/sj.ki.5001759 17024168

[35] Canaud B , Bragg‐Gresham JL , Marshall MR , et al. Response to ‘Mortality risk for patients receiving hemodiafiltration versus hemodialysis’. Kidney Int. 2006;70(8):1524‐1525. doi:10.1038/sj.ki.5001771 16641921

[36] Panichi V , Rizza GM , Paoletti S , et al. Chronic inflammation and mortality in haemodialysis: effect of different renal replacement therapies. Results from the RISCAVID study. Nephrol Dial Transplant. 2008;23(7):2337‐2343. doi:10.1093/ndt/gfm951 18305316

[37] See EJ , Hedley J , Agar JWM , et al. Patient survival on haemodiafiltration and haemodialysis: a cohort study using the Australia and New Zealand Dialysis and Transplant Registry. Nephrol Dial Transplant. 2019;34(2):326‐338. doi:10.1093/ndt/gfy209 30124954

[38] Kikuchi K , Hamano T , Wada A , Nakai S , Masakane I . Predilution online hemodiafiltration is associated with improved survival compared with hemodialysis. Kidney Int. 2019;95(4):929‐938. doi:10.1016/j.kint.2018.10.036 30782421

[39] Imamović G , Hrvačević R , Kapun S , et al. Survival of incident patients on high‐volume online hemodiafiltration compared to low‐volume online hemodiafiltration and high‐flux hemodialysis. Int Urol Nephrol. 2014;46(6):1191‐1200. doi:10.1007/s11255-013-0526-8 24057682

[40] Canaud B , Bayh I , Marcelli D , et al. Improved survival of incident patients with high‐volume haemodiafiltration: a propensity‐matched cohort study with inverse probability of censoring weighting. Nephron. 2015;129(3):179‐188. doi:10.1159/000371446 25765538

[41] Maduell F , Varas J , Ramos R , et al. Hemodiafiltration reduces all‐cause and cardiovascular mortality in incident hemodialysis patients: a propensity‐matched cohort study. Am J Nephrol. 2017;46(4):288‐297. doi:10.1159/000481669 29041011

[42] Policy and practice: the role real‐world data (RWD) in health technology assessment. 8.H. Workshop. 14th European Public Health Conference 2021; November 12, 2021. Eur J Public Health. 2021;31(suppl 3):ckab164.571. doi:10.1093/eurpub/ckab164.571

[43] Bolislis WR , Fay M , Kühler TC . Use of real‐world data for new drug applications and line extensions. Clin Ther. 2020;42(5):926‐938. doi:10.1016/j.clinthera.2020.03.006 32340916

[44] Kolasa KH , Huasen B . Has the time come to replace randomized controlled trials with real‐world evidence? A case of medical devices. Value Outcomes Spotlight. 2019;5(6):26‐29.

[45] Flynn R , Plueschke K , Quinten C , et al. Marketing authorization applications made to the European Medicines Agency in 2018‐2019: what was the contribution of real‐world evidence? Clin Pharmacol Ther. 2022;111(1):90‐97. 34689339 10.1002/cpt.2461 PMC9299056

[46] European Commission . Pharmaceutical Strategy for Europe. 2020. January 1, 2022. https://ec.europa.eu/health/system/files/2021-02/pharma-strategy_report_en_0.pdf

[47] Cogan K , Nolan L . The power of partnerships to realise the EU pharmaceutical strategy. Eurohealth. 2020;26(3):14‐17.

[48] Arlett P , Kjær J , Broich K , Cooke E . Real‐world evidence in EU Medicines regulation: enabling use and establishing value. Clin Pharmacol Ther. 2022;111(1):21‐23. doi:10.1002/cpt.2479 34797920 PMC9299492

[49] Kish J , Feinberg B , Hua D , et al. Use of real‐world evidence in clinical decision making by community oncologists. Value Health. 2018;21:S48. doi:10.1016/j.jval.2018.04.276

[50] Villines TC , Cziraky MJ , Amin AN . Awareness, knowledge, and utility of RCT data vs RWE: results from a survey of US cardiologists: real‐world evidence in clinical decision making. Clin Med Insights Cardiol. 2020;14:1179546820953410. doi:10.1177/1179546820953410 32952404 PMC7476349

[51] Katkade VB , Sanders KN , Zou KH . Real world data: an opportunity to supplement existing evidence for the use of long‐established medicines in health care decision making. J Multidiscip Healthc. 2018;11:295‐304. doi:10.2147/JMDH.S160029 29997436 PMC6033114

[52] Facey KM , Rannanheimo P , Batchelor L , Borchardt M , de Cock J . Real‐world evidence to support payer/HTA decisions about highly innovative technologies in the EU—actions for stakeholders. Int J Technol Assess Health Care. 2020;36(4):1‐10. doi:10.1017/S026646232000063X 32878663

[53] Hurwitz JT , Brown M , Graff JS , Peters L , Malone DC . Is real‐world evidence used in P&T monographs and therapeutic class reviews? J Manag Care Spec Pharm. 2020;26(12):1604‐1611. 33251991 10.18553/jmcp.2020.26.12.1604 PMC10391281

[54] Roberts MH , Ferguson GT . Real‐world evidence: bridging gaps in evidence to guide payer decisions. Pharmacoecon Open. 2021;5(1):3‐11. doi:10.1007/s41669-020-00221-y 32557235 PMC7895868

[55] Segal JB , Kallich JD , Oppenheim ER , et al. Using certification to promote uptake of real‐world evidence by payers. J Manag Care Spec Pharm. 2016;22(3):191‐196. doi:10.18553/jmcp.2016.22.3.191 27003547 PMC10397920

[56] Ostrovsky L . Payers' perspective: incorporating real‐world evidence in patient care. Am Health Drug Benefits. 2017;10(2):88‐90. 28626505 PMC5470246

[57] Deverka PA , Douglas MP , Phillips KA . Use of real‐world evidence in US payer coverage decision‐making for next‐generation sequencing‐based tests: challenges, opportunities, and potential solutions. Value Health. 2020;23(5):540‐550. doi:10.1016/j.jval.2020.02.001 32389218 PMC7219085

[58] H4RT Trial . The High‐volume Haemodiafiltration vs High‐flux Haemodialysis Registry Trial H4RT Trial. University of Bristol. Bristol Medical School: Population Health Sciences. December 10, 2021. https://www.bristol.ac.uk/population-health-sciences/projects/h4rt-trial/

[59] AlSahow A , Muenz D , Al‐Ghonaim MA , et al. *Kt/V*: achievement, predictors and relationship to mortality in hemodialysis patients in the Gulf Cooperation Council countries: results from DOPPS (2012‐18). Clin Kidney J. 2021;14(3):820‐830. doi:10.1093/ckj/sfz195 33777365 PMC7986324

[60] Canaud B , Vienken J , Ash S , Ward RA , Kidney Health Initiative HDF Workgroup . Hemodiafiltration to address unmet medical needs ESKD patients. Clin J Am Soc Nephrol. 2018;13(9):1435‐1443. doi:10.2215/CJN.12631117 29511057 PMC6140578

[61] Basile C , Davenport A , Blankestijn PJ . Why choose high volume online post‐dilution hemodiafiltration? J Nephrol. 2017;30(2):181‐186. 27586123 10.1007/s40620-016-0343-0

